# Taurine: A Source and Application for the Relief of Visual Fatigue

**DOI:** 10.3390/nu15081843

**Published:** 2023-04-12

**Authors:** Hao Duan, Wei Song, Jinhong Guo, Wenjie Yan

**Affiliations:** 1 College of Biochemical Engineering, Beijing Union University, Beijing 100023, China; 20221083210418@buu.edu.cn (H.D.);; 2 Beijing Key Laboratory of Bioactive Substances and Functional Food, Beijing Union University, Beijing 100023, China

**Keywords:** taurine, visual fatigue, source, safety, relief of visual fatigue

## Abstract

According to reports, supplementation with appropriate doses of taurine may help to reduce visual fatigue. Presently, some progress has been made in research related to taurine in eye health, but the lack of systematic summaries has led to the neglect of its application in the relief of visual fatigue. This paper, therefore, provides a systematic review of the sources of taurine, including the endogenous metabolic and exogenous dietary pathways, as well as a detailed review of the distribution and production of exogenous taurine. The physiological mechanisms underlying the production of visual fatigue are summarized and the research progress of taurine in relieving visual fatigue is reviewed, including the safety of consumption and the mechanism of action in relieving visual fatigue, in order to provide some reference basis and inspiration for the development and application of taurine in functional foods for relieving visual fatigue.

## 1. Introduction

When the eye is overused, or when the environment is too dry or disturbed by other physiological (and possibly pathological) factors, it is easy for the eye to adjust the refraction for a long time in order to form normal visual feedback, thus triggering visual fatigue, which is mainly manifested by frequent blurred vision, eye soreness, dryness, tearing and other eye discomfort [1]. A study in 2021 showed that the prevalence of visual fatigue among university students around the world is 46~71% [2]. Visual fatigue not only affects work and study status, but can also lead to video terminal syndrome and dry eye syndrome if one remains in a chronic state of visual fatigue [3]. At the same time, patients with eye diseases, such as strabismus and dry eye, are more likely to suffer from visual fatigue. It has been reported that 71.3% of people with dry eye suffer from varying degrees of visual fatigue [4]. At present, the incidence of visual fatigue is high and the number of people affected is increasing every year [5]. These people are of all ages and are, especially, students, teachers and those who work with electronic devices such as computers [6,7,8]. This shows that visual fatigue occurs in all age groups and that the number of people who need relief from visual fatigue is very large. Studies have shown that the physiological mechanisms of visual fatigue are mainly due to various stress injuries to the retina or macula and damage to nerve cells.

### 1.1. Retinal Stress Damage: Oxidation, Inflammation, Apoptosis

The retina, located in the fundus of the eye, is a fragile neuronal tissue that perceives and receives external light to form visual signals that are transmitted to the brain to obtain external information [9]. The retina is composed of cells such as the pigment epithelium (RPE), optic cells, bipolar cells and ganglion cells, and studies have shown that reduced retinal function is one of the major causes of the development of visual fatigue [10]. In particular, impairment of the structural integrity and function of the RPE will directly affect retinal function [11]. RPE, as one of the most metabolically active tissues within the body, has a high demand for oxygen and is, therefore, susceptible to the production of reactive oxygen species (ROS) and their attack. When the eye is exposed to prolonged work, intense or dense light and high oxygen pressure, the metabolic rate of the extraocular and ciliary muscles increases, contributing to the accumulation of large amounts of metabolites such as peroxides and ROS. When there is an imbalance between the production of free radicals and endogenous antioxidant defense mechanisms, this results in oxidative stress damage to the eye [12], leading to the development of visual fatigue. At the same time, there are large amounts of polyunsaturated fatty acids (PUFA) in the retina, particularly docosahexaenoic acid, which are essential for the integrity of photoreceptor cells and are involved in the phototransduction cascade [13]. ROS tend to oxidize these PUFA, producing large amounts of lipid peroxides and further inducing the production of large amounts of reactive aldehydes, such as 4-hydroxynonenoic acid and Malondialdehyde (MDA), which, when bound to intracellular proteins and DNA, can cause cellular inflammation and apoptosis [14], thereby inducing visual fatigue.

The aging of RPE will lead to the aging of the eyes, the reduction of macular pigment optical density (MPOD) and the impairment of macular function, affecting the normal visual function. Furthermore, excessive accumulation of N-retinylidene-N-retinylethanolamine (A2E) on the RPE predisposes cells to oxidative stress or cell death, a condition that can also contribute to the development of visual fatigue [15]. Li et al. [16] found that the intervention of a 28 g wolfberry diet 5 times per week for 90 days in healthy people can significantly increase the retinal eccentrics of MPOD at 0.25 and 1.75, indicating that regular intake of wolfberry in healthy middle-aged people can increase MPOD, which is helpful to prevent or delay macular injury. This effectively protects the retina from the effects of aging. From another perspective, it also suggests that dietary interventions exerting retinal protection strategies are feasible.

### 1.2. Damage to Nerve Cells

The retina is rich in various nerves and retinal glial cells include microglia, astrocytes and retinal Müller cells. Among them, the retinal macroglia (Müller cells and astrocytes) and microglia are very important in maintaining retinal homeostasis and protecting the retina from damage [17,18]. Studies have shown that retinal macroglia are the basis for maintaining retinal neuronal homeostasis [19]. Müller cells, which have the highest content—up to 90%—are the main structural and nutritional support for retinal cells [20]. Astrocytes, on the other hand, play a role in protecting the retina from damage, mainly through the reactive glial proliferation pathway [21]. However, increased levels of some proteins expressed by reactive astrocytes, such as Matrix metalloproteinase-9 (MMP-9) and Urokinase-type Plasminogen Activator (uPA), can cause retinal damage [22], so the role of astrocytes in the retina is dual. In contrast, retinal microglia play a deleterious role in cell death [23]. When microglia are activated, this leads to an accelerated release of pro-inflammatory and pro-phagocytic factors, resulting in cell death and more microglia activation, which in turn contributes to inflammatory damage to the retina and photoreceptor damage [24]. At the same time, retinal glial cells are also involved in glutamate buffering in neurons. Glutamate is thought to be the main chemical signal in photoreceptors, bipolar cells and ganglion cells in the retina [25,26]. However, when glutamate concentrations are too high, it can cause neuronal excitotoxicity, with the result that neurons are damaged or even die [27]. When retinal Müller cells are damaged, the excitotoxicity of glutamate may be further exacerbated. It has been reported that the inflammatory damage condition of the retina, as well as the damaged photoreceptor condition, can be effectively protected by inhibiting microglia activity [28]. Thus, the regulation of glutamate homeostasis by retinal glial cells may exert an important protective mechanism against neuronal damage [29].

Taurine, chemically known as 2-aminoethanesulfonic acid, is a specific non-protein amino acid that is present in high concentrations in the brain, retina and muscle in its free state [30]. Data show that the levels of taurine in human brain, retina and plasma are 1~20 μmol/g, 30~40 μmol/g and 50~100 μmol/L, respectively [31]. Taurine is mainly involved in physiological activities, including bile acid binding, osmoregulation, neuronal excitability, inflammatory response and glucose metabolism [32]. For eye health, taurine plays an important role in promoting retinal differentiation and development [33], while lack of taurine in diet can lead to impaired retinal function and even blindness [34]. It has been reported in the literature that taurine depletion and exposure to light causes shortening of the outer segments of photoreceptor cells, activation of microglia, oxidative stress in the outer and inner nuclear layers and ganglion cell layer, loss of synapses [35] and loss of optic cone cells [36]. This suggests that taurine depletion can cause damage to cells such as retinal pigment epithelial cells, photoreceptors and retinal ganglion cells in the retina, contributing to increased glial cell responses and oxidative stress. It is also suggested that taurine is essential for ocular health and that dietary supplementation of taurine can effectively improve ocular stress damage and exert good neuroprotective effects, thereby repairing damaged retinas and reducing visual fatigue [37]. At present, some progress has been made in research related to taurine in eye health, but the lack of systematic summary has led to the neglect of its application in the alleviation of visual fatigue. This paper, therefore, provides a systematic review of the sources of taurine, including the endogenous metabolic and exogenous dietary pathways, as well as a detailed review of the distribution and production of exogenous taurine. The physiological mechanisms underlying the production of visual fatigue are summarized and the research progress of taurine in relieving visual fatigue is reviewed, including the safety of consumption and the mechanism of action in relieving visual fatigue, in order to provide some reference basis and inspiration for the development and application of taurine in functional foods for relieving visual fatigue.

## 2. Sources of Taurine

Currently, taurine is obtained from two sources: the endogenous metabolic synthetic pathway of the body and the exogenous dietary route.

### 2.1. Endogenous Metabolic Synthesis of Taurine

**Figure 1:**
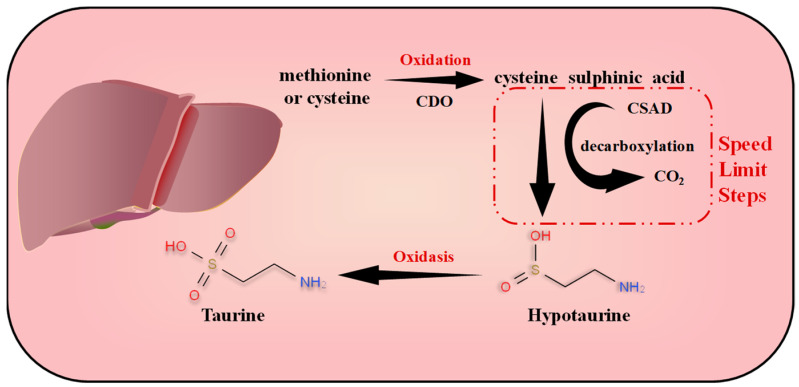
Taurine synthesis pathway in the liver. CDO = cysteine dioxygenase; CSAD = cysteine sulfinic acid decarboxylase.

Taurine is mainly found in the liver, central nervous system, retina, heart, skeletal muscle and other excitable tissues [38,39]. In mammals, taurine is synthesized from methionine or cysteine in the presence of adequate levels of vitamin B6 [40]. The pathway of taurine synthesis in the liver is detailed in Figure 1. In the endogenous synthesis of taurine, the decarboxylation reaction is the rate-limiting step and cysteine sulphinic acid decarboxylase (CSAD) and cysteine dioxygenase (CDO) are rate-limiting enzymes. Studies have shown that CSAD levels vary considerably between organisms, with higher CSAD activity in rodents and lower in humans, which seems to explain why cats prefer to prey on rats. Therefore, the normal requirements of the body cannot be met by the body’s own synthetic taurine content alone and a dietary supplement of exogenous taurine is required [41].

### 2.2. Exogenous Distribution and Mode of Production of Taurine

#### 2.2.1. Sources and Distribution

**Figure 2:**
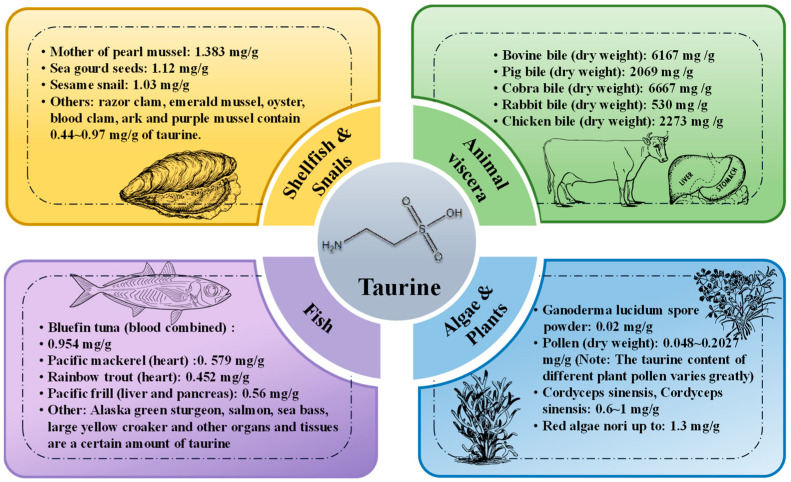
Main dietary sources of taurine and the proportion of taurine in each food [43,44,45,46].

It is reported that the main dietary sources of taurine are animal foods, especially marine organisms and mammals, which contain high concentrations of taurine in their organs and tissues [42]. In addition, seaweeds such as kelp and spirulina and a few plants such as Ganoderma lucidum spores, wolfberries and cordyceps also contain some taurine [43,44,45,46]. The main dietary sources of taurine and the proportion of each food are detailed in Figure 2.

#### 2.2.2. Mode of Production of Exogenous Taurine

Currently, exogenous taurine is produced by natural extraction, chemical synthesis and bio-fermentation and each production method has certain characteristics, as detailed in Table 1.

##### Extraction Methods

Taurine is soluble in water, but insoluble in ethanol, ether and acetone. Boiling is a simple and inexpensive method of extraction. However, the long extraction time and low extraction efficiency of the boiling method have been observed in the early years of extraction research [47]. Currently, the boiling method is mostly supplemented by other extraction methods to compensate for the shortcomings of a single extraction, e.g., ultrasound-assisted boiling extraction [48]. Ultrasound-assisted extraction is a new non-thermal physical processing technology that uses ultrasound cavitation to disrupt cell membranes (walls), in order to accelerate and enhance the dissolution of active ingredients in natural products and, therefore, induce a certain solubilizing effect [49]. Compared to conventional extraction methods, ultrasonic extraction has the advantages of suitability for all kinds of solvents, fast extraction speed, simple method, high yield and reduced solvent consumption, and it is particularly suitable for the extraction of heat unstable components [50,51]. However, ultrasonic extraction techniques are difficult and costly to prepare for industrial implementation. Therefore, it is mainly used for laboratory studies.

Solvent extraction is also an extraction method for taurine from natural products. A solution of 60–75% ethanol is mostly used as a solvent for extraction in existing studies, but the extraction rate of taurine is not greatly improved compared to boiling [52,53,54] and ethanol as an extraction solvent for industrial production is not only costly, but also poses certain safety risks.

Enzymatic digestion can also be used for the extraction of taurine and can be combined with boiling. Enzymatic digestion has the advantages of safe operation, high extraction efficiency and low cost, but the experimental conditions are strict and the appropriate enzyme type needs to be determined according to the target. Enzymes have a high degree of specificity, the source of raw materials is different and the corresponding enzyme hydrolysis types need to be investigated. Taurine is mostly sourced from animal foods and the use of direct hydrolysis by biological enzymes can result in the dissolution of free hydrogenated acids, peptides and trace elements from animal raw materials in the enzymatic digest, leading to increased difficulty in separation [55]. In addition to boiling, solvent extraction, enzymatic digestion and ultrasound-assisted methods, there are also ultra-high pressure treatment methods [60] or a combination of extraction methods to extract taurine. In general, the extraction efficiency and effectiveness of taurine from natural products are poor if a single extraction method is used. Therefore, a combination of extraction methods has been used to improve the extraction efficiency and yield. Additionally, the cost of obtaining taurine from natural extraction is high and most of these methods are not suitable for industrial production.

The yield of taurine prepared by different extraction methods is detailed in Table 2.

##### Chemical Synthesis and Bio-Fermentation Methods

More than 20 methods have been developed for the chemical synthetic production of taurine. Of these, most are produced commercially by ethylene oxide or monoethanolamine [66]. However, ethylene oxide is more toxic, has higher reagent recovery costs and is also volatile and explosive. The production of taurine by this method is therefore very demanding and costly [56]. The industrial production of taurine from monoethanolamine has better safety and environmental benefits than the ethylene oxide process; it is a two-step process that involves the synthesis of 2-aminoethyl sulphate from monoethanolamine and sulphuric acid by heat treatment and the production of taurine from 2-aminoethyl sulphate by heat treatment with an acidic solution of sodium sulphite [57]. However, the production of taurine from monoethanolamine takes too long and consumes too much energy. Moreover, some intermediate products are susceptible to thermal hydrolysis, which can lead to an increase in by-products and affect the yield of taurine [58]. In conclusion, each method has its own advantages and disadvantages and each step of the reaction must be strictly controlled during the industrial production process.

In addition, bio-fermentation can also be used to produce taurine. At present, the following microorganisms have been reported for the production of taurine by bio-fermentation: *Yarrowia lipolytica*, *Chlamydomonas reinhardtii*, *Saccharomyces cerevisiae*, *Corynebacterium glutamicum*, etc. [59]. Among them, *Corynebacterium glutamicum* is considered to be a safe and suitable strain for taurine production. By constructing a recombinant strain of *Corynebacterium glutamicum*, 62.0 ± 1.5 mg/g (cell dry weight) of taurine can be obtained with high yield and low production cost [59], thus having a high industrial production value.

## 3. Research Progress on Taurine in the Relief of Visual Fatigue

### 3.1. Food Safety

The first reports on the safety of taurine for consumption began in 1999, when the Scientific Committee on Food (SCF) first evaluated the safety of ‘energy’ drinks containing taurine and concluded that taurine was not genotoxic, teratogenic nor carcinogenic [67]. More recently, Serrano et al. [68] have refined the data on the genotoxicity of taurine and their final results show that taurine did not show mutagenicity or genotoxicity in the Ames test, the micronucleus test nor the standard and enzyme-modified comet tests. The results of these toxicity studies on taurine confirm that it is not genotoxic, carcinogenic nor teratogenic [69,70,71,72]. Also, studies addressing taurine tolerance limits have been reported evaluating taurine no observable adverse effect levels (NOAEL). There was also a risk assessment study in human clinical trials that reported that no adverse effects were observed in subjects given a maximum daily dose of 3 g/d of taurine [73]. Furthermore, the European Food Safety Authority (EFSA) opinion concluded that taurine added to energy drinks at a dose of 1000 mg/kg/day was the NOAEL [69].

In addition, toxicological evaluation tests with taurine compounding also reflect the good safety of taurine for consumption. Wang Xue et al. [74] evaluated the toxicological safety of taurine as the main ingredient of the compound powder (daily consumption of each ingredient: taurine 0.91 g/d, inositol 32 mg/d, vitamin E 100 mg/d) and showed that SPF-grade Kunming breed mice undergoing an acute toxicity test were judged to exhibit maximum tolerated dose (MTD) > 20.0 g/kg·bw. In the 30-d feeding test in SD rats, no abnormal changes were observed in the overall health status, physiological and biochemical functions and organ histomorphology of the rats; the results of the mouse bone marrow multi-stained erythrocyte micronucleus test, sperm malformation test and rat Ames test were uniformly negative, indicating that the taurine powder was safe, non-toxic, non-mutagenic and sub-chronic toxic. Similarly, the toxicological evaluation of a beverage made from taurine with L-carnitine, D-hydroglucose hydrochloride, caffeine, inositol and B-vitamins was carried out and the final result was found to be non-toxic [75]. In addition, the toxicological evaluation of taurine in combination with selenium-enriched yeast or with lacto-mineral salts showed that the substance was safe and non-toxic [76,77].

These results show that taurine, a naturally occurring amino acid, has low toxicity.

### 3.2. Progress in Research on the Mechanism of Action of Taurine in Relieving Visual Fatigue

**Figure 3:**
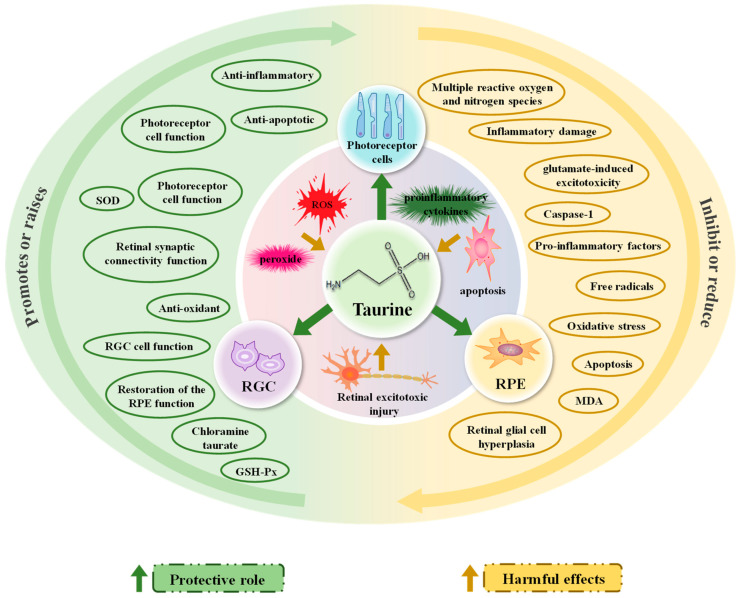
The main mechanisms by which taurine protects the retina to reduce visual fatigue. Taurine protects photoreceptor cells, RGC and EPE cells from damage caused by oxidative stress, inflammation, apoptosis and ocular neurotoxicity through various pathways (reducing adverse effects or promoting beneficial effects), thereby reducing visual fatigue.

Normal retinal function is essential for maintaining proper visual function and eye health. Therefore, ways to protect and repair damaged retinal function can help alleviate the onset of visual fatigue. The main mechanisms by which taurine protects the retina to reduce visual fatigue are detailed in Figure 3.

#### 3.2.1. Reducing Retinal Stress Damage: Oxidation, Inflammation, Apoptosis

The main cause of visual fatigue is the continuous work of the eye or the exposure of the eye to strong light and oxygen, a situation that promotes enhanced ocular metabolism followed by an increase in peroxides and, therefore, vulnerability to oxidative damage, which begins to occur [78]. Current studies have shown that one of the most important protective effects of taurine on cells is its antioxidant effect, mediated by three different processes: First, taurine neutralizes the neutrophil oxidant hypochlorous acid and the resulting reaction product, chloramine taurate, is also better at hindering the onset of inflammation [79,80]. Second, taurine reduces superoxide production by mitochondrial metabolism [81,82]. Third, ROS produced by cellular metabolism or external environmental stimuli tend to attack antioxidant enzymes, leading to increased oxidative damage [82,83], whereas taurine can effectively protect these antioxidant enzymes from ROS attack, thus counteracting oxidative stress [33,84]. It is well known that light exposure tends to lead to retinal stress damage and damage or loss of photoreceptors and dietary taurine supplementation can increase the concentration of taurine in the retina that is reduced by light exposure, reduce light exposure-induced retinal MDA overproduction and increase retinal superoxide dismutase (SOD) and glutathione peroxidase (GSH-Px) activity in the retina. At the same time, taurine also inhibited caspase-1 expression in the apoptotic signaling pathway of photoreceptor cells, suggesting that dietary taurine may reduce photochemical stress-induced retinal damage by mediating retinal antioxidant and anti-apoptotic mechanisms, which also suggests that taurine has an indispensable and important physiological role in the structural and functional development of the retina [85]. In a study by Diego et al. [86], it was again confirmed that taurine is an essential nutrient for the maintenance of normal physiological activity of retinal cells, especially in the presence of light-induced photoreceptor damage, and that the retina has a higher requirement for taurine. Moreover, taurine significantly improved taurine levels and photoreceptor survival in retinal dystrophy rats, reduced the release of pro-inflammatory factors and oxidative stress damage in the retina and effectively repaired and reduced RPE cell damage [87]. This suggests that taurine may reduce damage to retinal function through anti-inflammatory and antioxidant pathways [88]. In addition, taurine can also prevent retinal and optic nerve morphological changes by reducing the retinal oxidative stress pathway and inhibiting Endothelin-1 (a potent vasoconstrictor involved in glaucomatous retinal vascular dysregulation and oxidative stress)-induced retinal cell apoptosis [89].

Studies have shown that the visual function of the eye declines with age. Retinal damage in some animal models of ageing may also be closely related to taurine deficiency [90]. Wang et al. used targeted metabolomics to analyze and compare the metabolites in the eyes of 6-week-old C57 BL6/J young mice with those of 73-week-old aged mice. The results of the assay showed that senescent mice had reduced electroretinogram responses and reduced number of photoreceptors compared to young mice, and impaired taurine metabolism was observed [91]. In vivo and ex vivo experimental studies have shown that taurine ameliorates AMD damage by reducing the retinal apoptotic pathway [92].

The above studies have shown that taurine has a very important role in maintaining normal visual function as well as photoreceptor function. Taurine can effectively improve stress damage, especially oxidative stress damage, arising in the retina. In addition, taurine can also play a role in protecting retinal function from stress damage through anti-inflammatory and anti-apoptotic effects, thus relieving visual fatigue.

#### 3.2.2. Reducing Retinal Excitotoxic Damage and Providing Neuroprotection

The bipolar cells, photoreceptors and ganglion cells of the eye contain high concentrations of glutamate, an important neurotransmitter, which, when overloaded, triggers excitotoxicity and apoptotic signaling, leading to retinal dysfunction [93]. Furthermore, retinal excitotoxic damage is also the basis for loss of visual function [94,95]. This retinal excitotoxic damage also includes overstimulation of glutamate receptors, especially the N-methyl-D-aspartic acid (NMDA) subtype of glutamate receptors, which causes calcium inward flow and disrupts intracellular environmental homeostasis, triggering pro-apoptotic pathways [96]. Therefore, induction of retinal damage in rats by intravitreal injection of NMDA has been used as a representative animal model of excitotoxic retinal damage [97]. In contrast, taurine has been shown to provide neuroprotection through a pathway that reduces intracellular free calcium, thereby exerting resistance to glutamate-induced excitotoxicity [98]. Also, taurine is a potential neuromodulator of glutamate transmission [99]. Taurine can effectively inhibit glutamate-induced excitotoxicity and protect retinal function from damage through long-term dietary supplementation of the taurine pathway [100]. In addition, experimental data show that taurine is effective in scavenging a wide range of reactive oxygen and nitrogen species at different physiological concentrations [101]. Therefore, the reducing effect of dietary taurine on retinal oxidative stress may not only be due to its ability to restore and strengthen the retinal antioxidant defense, but also due to taurine’s ability to enhance free radical bursting and thus prevent NMDA-induced retinal damage by reducing retinal oxidative stress [102].

The Royal College of Surgeons (RCS) rat is a commonly used animal model for retinitis pigmentosa. The main manifestations of this rat are impaired phagocytosis of RPE cells [103,104,105,106] with progressive photoreceptor degeneration [107,108,109,110], increased retinal glial cell proliferation, altered retrograde axonal transport of retinal ganglion cells (RGC) and loss of RGC [111,112,113]. One study claimed that the first sign of retinal degeneration in RCS rats was a significant decrease in taurine levels. Data show that retinal taurine is supplied by RPE cells and Müller cells [91,114]. At the same time, taurine depletion also increases retinal glial cell proliferation and impairs the phagocytosis of RPE cells [35]. This suggests that taurine is indispensable for maintaining normal RPE function and that ameliorating the detrimental damage to RPE cells by taurine supplementation may be a potential dietary intervention strategy. Indeed, the addition of 0.2 M taurine to the drinking water of RCS rats revealed a significant reduction in the number of microglia in the outer retinal layer, an effective reduction in glial fibrillary acidic protein (GFAP) content in Müller cells, a significant reduction in oxidative stress in the outer and inner retinal nuclei and also improved maintenance of retinal synaptic connections. This suggests that dietary taurine may act to improve photoreceptor damage and increase retinal electrical responses by mediating various neuroprotective mechanisms [88].

In addition, taurine may also play a role in improving retinal damage by improving retinal synaptic connections, maintaining the balance of the Bcl-2/Bax ratio (which determines apoptosis) and reducing reactive glial proliferation in retinal Müller cells [115]. In conclusion, the above studies suggest that taurine may play a role in improving the normal function and morphology of the damaged RPE, RGC and photoreceptors by reducing excitotoxicity in the retina as well as a variety of neuroprotective mechanisms, thereby alleviating visual fatigue.

The health effects of taurine on the retina and its necessity are detailed in Table 3.

## 4. Conclusions

Visual fatigue is mainly caused by a decline or impairment of retinal function, especially when prolonged eye use leads to an excessive accumulation of metabolic waste in the eye that cannot be cleared, causing various stress injuries. Taurine is considered an essential nutrient for the function and survival of retinal photoreceptors, RGCs and RPE cells [36,87,116], and its depletion increases the susceptibility of the retina to light exposure damage [35,36] and also increases glial cell proliferation and oxidative stress in the retina, impairing the normal phagocytic capacity of the RPE [35]. In addition, numerous clinical [117,118] and animal studies [33,119,120] have shown that taurine depletion can induce impaired retinal function and even pathological eye disease. Taurine supplementation is effective in improving retinal photoreceptor degeneration [88,121] and retinal and optic nerve damage [33,90]. However, the current research on the protective effect of taurine on the retina is mainly focused on its antioxidant effect and research on non-antioxidant pathways to improve retinal damage may be an important direction for future research. In addition, taurine, as a natural amino acid, has a high safety profile and is allowed to be added to functional drinks and other food products in many countries. Due to the important protective role of taurine in the retina and its high food safety, its application in the development of functional foods for the relief of visual fatigue is very promising. Therefore, in the future development of functional foods for the relief of visual fatigue, research on the synergistic effect of taurine in combination with other functional ingredients can be enhanced to clarify the optimal formulation ratio between taurine and other functional ingredients, so as to maximize the visual fatigue relief function of the products.

A rational selection and extraction study of raw materials is one of the necessary and effective means to maximize their function in formulation. Therefore, this paper systematically reviews the endogenous synthesis pathways and exogenous dietary distribution of taurine in the human body, as well as its extraction and industrial production. Both chemical synthesis and bio-fermentation methods are suitable for the industrial production of taurine of high purity, but each has certain disadvantages. Extraction methods, on the other hand, are not suitable for the industrial production of higher purity taurine, but are of great importance for the development of functional food ingredients. This is because the body obtains taurine through exogenous means by consuming a certain dose of taurine-rich food, which is then digested, absorbed and metabolized by the body in order to truly replenish the body with the required amount of taurine. This process emphasizes not only the level of taurine content in food, but also the relationship between dose and efficacy. The selection of suitable raw materials and extraction methods can effectively increase the content of taurine in raw materials and reduce the dosage of the raw materials themselves, which improves the utilization of raw materials, enriches the range of raw materials available for the formulation of functional foods for the relief of visual fatigue, improves the novelty of product formulations and also facilitates researchers to diversify the design of functional food dosage forms according to the characteristics of the population. It is also very suitable for the research and development of new food ingredients as well as functional food ingredients.

In conclusion, taurine has an important protective effect on retinal function, mainly due to its antioxidant and neuroprotective effects, and is essential for the function and survival of retinal photoreceptors, RGC and RPE cells. However, as the amount of taurine synthesized by the human body is not sufficient to meet the health needs of the body, it needs to be obtained through dietary supplementation. This suggests the importance and necessity of dietary taurine supplementation in promoting ocular health. This paper, therefore, provides a systematic review of the sources of taurine, including the endogenous metabolic and exogenous dietary routes, as well as a detailed review of the distribution and production of exogenous taurine. The physiological mechanisms underlying the production of visual fatigue are summarized and the research progress of taurine in relieving visual fatigue is reviewed, including the safety of consumption and the mechanism of action in relieving visual fatigue, with a view to providing some reference and inspiration for the development and application of taurine in functional foods for relieving visual fatigue.

## Figures and Tables

**Table 1:** Characteristics of different production methods of taurine.

Production Methods	Scale of Production	Advantages	Disadvantages	Ref.
Extraction method	Study of extract-based ingredients suitable for functional foods	The extraction and development of natural products are very important for functional food	It is not suitable for industrial production of taurine because of its high cost and low efficiency	[47,48,49,50,51,52,53,54,55]
Chemical synthesis	Suitable for industrial mass production	Suitable for the production of large quantities of taurine	Chemical reagents are used and safety needs to be carefully considered	[56,57,58]
Bio fermentation	Suitable for industrial mass production	Suitable for producing large quantities of taurine in a safe and cost-effective manner	The type of enzymatic digestion used needs to be determined	[59]

**Table 2:** Yields of different extraction methods of taurine.

No.	Withdrawal Method	Extraction Objects	Optimal Extraction Process Parameters	Achievement Rate	Ref.
1	Solvent extraction	Scapharca broughtonii	Material–liquid ratio 1:12 (g/mL), 75% ethanol by volume, extraction time 50 min, temperature 70 °C	5.36 mg/g	[52]
2	Solvent extraction	Perna viridis	Extraction temperature 80 °C, ethanol concentration 60%, extraction time 60 min, material to liquid ratio 1:5 (g/mL), extraction times 3 times	9.20 mg/g	[53]
3	Ultrasound-assisted enzymatic extraction	Mussel Meat	Extraction enzyme: papain; enzyme addition 4374 U/g, sonication time 19 min, sonication power 200 W, enzymatic digestion time 3 h	11.21 mg/g	[60]
4	Enzymatic digestion	Mussel Meat	Extraction enzyme: papain; enzymatic digestion temperature 50 °C, enzyme addition 4000 U/g, enzymatic digestion time 3 h, enzymatic digestion pH 7.5	10.74 mg/g	[60]
5	UHP-assisted enzymatic extraction	Mussel Meat	Extraction enzyme: papain; pressure 200 MPa, holding time 3 min, enzymatic digestion time 3.5 h and enzyme addition 5000 U/g	11.63 mg/g	[60]
6	High voltage pulsed electric field assisted enzymatic digestion	Mussel Meat	Extraction enzyme: papain; electric field strength 25 kV/cm, number of pulses 10, enzymatic digestion time 2.95 h	13.77 mg/g	[60]
7	ultrasonic-assisted water extraction	aquatic shellfish (*Pinctada martensii meat*)	Ultrasonic power 240 W, ultrasonic time 70 min, water extraction temperature 70 °C, water extraction time 2 h, material to liquid ratio 1:4 (g/g)	7.155 ± 0.04 mg/g	[61]
8	ultrasonic-assisted water extraction	bovine liver	Ultrasonic power of 205 W, ultrasonic time of 12.18 min, liquid to material ratio of 1:4 (mL/g)	6.17 ± 0.17 mg/g	[62]
9	ultrasonic-assisted water extraction	red algae *Porphyra yezoensis*	Ultrasonic power of 300.0 W, extraction time of 38.3 min, extraction temperature of 40.5 °C	13.0 mg/g	[63]
10	Enzymatic digestion	ruditapes philippinarum	Alkaline protease was the most suitable enzyme for the extraction; the enzymatic digestion temperature was 45 °C, the digestion time was 3.5 h and the pH was 8.0.	3.08 mg/g	[64]
11	Enzymatic digestion	Oyster Meat	Extraction enzyme: neutral protease; 1300 U/g, pH 7.5, temperature 48 °C	2.724 mg/g	[65]

**Table 3:** Health effects of taurine on the retina and the need for it.

No.	Animal/Cell	Experimental Models	Intervention Pathways	Dose	Periodicity	Experimental Results	Conclusions/Potential Mechanisms	Ref.
1	SD rats	glial cell activation and oxidative stress induced by taurine deficiency secondary to β-alanine administration and light exposure	3% β-alanine in drinking water (taurine depleted)	--	2 m	(1) TAU depletion caused a decrease in retinal thickness, shortening of photoreceptor outer segments, microglial cell activation, oxidative stress in the outer and inner nuclear layers and the ganglion cell layer and synaptic loss. (2) These events were also observed in light exposed animals, which in addition showed photoreceptor death and macroglial cell reactivity. (3) Light exposure under taurine depletion further increased glial cell reaction and oxidative stress. (4) The retinal pigment epithelial cells were Fluorogold labeled and whole-mounted and we document that taurine depletion impairs their phagocytic capacity.	TAU depletion causes cell damage to various retinal layers including retinal pigment epithelial cells, photoreceptors and retinal ganglion cells, and increases the susceptibility of the photoreceptor outer segments to light damage.	[35]
2	Albino SD rats	β-alanine supplementation induces taurine depletion	β-alanine supplementation (3%) in the drinking water	--	2 m	(1) β-alanine supplementation induces TAU depletion and causes alterations of the retinal nerve fiber layer and axonal transport by retinal ganglion cells.	TAU depletion causes RGC loss and axonal transport impairment	[36]
3	SD rats	retinal damage produced by photochemical stress	Blended into feed	4 g taurine/100 g diet	15 d	(1) Dietary TAU is effective in preventing morphological changes in the retina from photochemical damage. (2) Increased TAU content, decreased MDA content and increased SOD and GSH-Px activity in the retina. (3) Dietary TAU inhibited activator protein-1 (AP-1) (c-fos/c-jun subunits) expression, up-regulated NF-kB(p65) expression and decreased caspase-1 expression so as to reduce the apoptosis of photoreceptors in the retina.	Dietary TAU attenuates photochemical stress-induced retinal damage through antioxidant and antiap-1-NF-kB-caspase-1 apoptotic mechanisms.	[85]
4	albino SD rats	β-alanine in the drinking water to induce taurine depletion + light- induced photoreceptor degeneration	Drinking water added	--	2 m	(1) Light exposure did not affect the numbers of Brn3a^+^RGCs or m^+^RGCs but diminished the numbers of S- and L/M-cones and caused the appearance of rings devoid of cones, mainly in an “arciform” area in the superotemporal retina. (2) Light exposure under taurine depletion increased photoreceptor degeneration but did not seem to increase Brn3a^+^RGCs or m^+^RGCs loss.	TAU is essential for the survival of rat retinal cells, especially under light-induced photoreceptor degeneration. TAU supplementation may help prevent retinal damage.	[86]
5	RCS rats	RCS rats suffering retinal degeneration secondary to impaired retinal pigment epithelium phagocytosis caused by a MERTK mutation	Drinking water added	2 mol/L	24 d	(1) TAU increases taurine plasma levels and photoreceptor survival in rats. (2) Electroretinograms showed increases of 70% in the rod response, 400% in the a-wave amplitude, 30% in the b-wave amplitude and 75% in the photopic b-wave response in treated animals. Electroretinograms showed increases of 70% in the rod response, 400% in the a-wave amplitude, 30% in the b-wave amplitude and 75% in the photopic b-wave response in treated animals. (3) Animals in the TAU intervention group had a reduced number of microglia in the outer retinal layer, reduced GFAP expression in Müller cells, reduced oxidative stress in the outer and inner nuclear layers and improved maintenance of synaptic connections. (4) Increased FG phagocytosis in retinal pigment epithelial cells of animals in the taurine intervention group.	TAU reduces photoreceptor damage in dystrophic RCS rats. Increases the electrical response of the retina and these effects may be mediated through various neuroprotective mechanisms.	[87]
6	SD rats	ET-1 induced retinal and optic nerve damage	received an intravitreal injection	320 nmol/L	7 d	(1) TAU has a significant protective effect against ET-1-induced retinal and optic nerve damage. (2) Based on morphological observations, caspase immunostaining showed a significant reduction in the number of apoptotic retinal cells in the TAU pretreatment group. (3) Retinal oxidative stress was reduced in all TAU intervention groups.	TAU prevents ET-1-induced apoptosis in retinal cells and the protective effect on ET-1-induced retinal and optic nerve damage is associated with reduced retinal oxidative stress.	[89]
7	retinal neuronal cells	blue light-induced apoptosis in retinal neuronal cells	in vitro	--	--	(1) Blue light increased osmolyte transporter mRNA expression together with osmolyte uptake. (2) TAU significantly suppressed blue light-induced retinal neuronal cell apoptosis.	The compatible osmolyte taurine may have an important role in cell resistance to blue light and cell survival.	[92]
8	Cultured Neurons	Glutamate-induced Apoptosis in Cultured Neurons	in vitro	--	--	(1) TAU inhibits glutamate-induced down-regulation of Bcl-2 and up-regulation of Bax Glutamate-induced apoptosis is dependent on calpain activation and TAU inhibits glutamate-induced calpain activation.	The antiapoptotic function of TAU is due to its inhibition of glutamate-induced membrane de- polarization.	[98]
9	SD rats	injected with streptozotocin to establish experimental diabetic model	Feed adulteration	1.2% taurine feed	4–12 W	(1) Dietary TAU supplementation is effective in improving the histopathological and ultrastructural changes in diabetic retinopathy. (2) Dietary taurine supplementation can increase TAU levels and decrease glutamate and aminobutyric acid levels in the diabetic retina. (3) TAU supplementation increased glutamate transporter (GLAST) expression, decreased intermediate filament glial fibrillary acidic protein (GFAP) and N-methyl-D-aspartate receptor subunit 1 (NR1) expression in diabetic retina.	Chronic TAU supplementation improves retinopathy in diabetic rats through a pathway that counteracts the excitotoxicity of glutamate.	[100]
10	SD rats	NMDA-induced retinal damage	received an intravitreal injection	--	7 d	(1) Treatment with TAU, particularly pre-treatment, significantly increased retinal glutathione, SOD and catalase levels compared to NMDA-treated rats. The levels of MDA reduced significantly. (2) Reduction in retinal oxidative stress in TAU pre-treated group was associated with significantly greater fractional thickness of ganglion cell layer within inner retina and retinal cell density in inner retina. (3) TUNEL staining showed significantly reduced apoptotic cell count in TAU pre-treated group compared to NMDA group.	TAU protects against NMDA-induced retinal injury in rats by reducing retinal oxidative stress.	[102]
11	SD rats	STZ-induced diabetic rats	intraperitoneal injection or by intragastric administration	420 mg/kg(wt)/d by intraperitoneal injection or 1 mL/100 g(wt)/d by gavage in drinking water at a concentration of 0.5 mol/L	4 W	(1) TAU significantly prevented the reduction of photopic b-wave amplitude and retinal cone cells and ganglion cells loss and maintained the Bcl-2/Bax ratio balance in diabetic rats. TAU also prevented the upregulation of glial fibrillary acidic protein (GFAP) and reduced retinal reactive gliosis (2) TAU reduced plasma glutamate and tyrosine levels, which were elevated in diabetic rats. (3) mGluR6 levels reduction detected by western blot and immunofluorescence in diabetic retinas was inhibited and the displacement of mGluR6 in OPL into the inner nuclear layer (INL) detected by immunofluorescence was reduced by TAU treatment.	TAU may protect retinal cells from diabetic attacks by activating Tau-T, reducing retinal reactive gliosis, improving retinal synaptic connections and decreasing retinal cell apoptosis.	[115]

SD = Sprague-Dawley; RCS = Royal College of Surgeons; TAU = Taurine; MDA = Malondialdehyde; SOD = Superoxide Dismutase; GSH-Px = Glutathione peroxidase; GFAP = glial fibrillary acidic protein; ET-1 = Endothelin-1; ET-1 = Endothelin-1.

## Data Availability

Not applicable.
